# Classification of Rice Heavy Metal Stress Levels Based on Phenological Characteristics Using Remote Sensing Time-Series Images and Data Mining Algorithms

**DOI:** 10.3390/s18124425

**Published:** 2018-12-14

**Authors:** Tianjiao Liu, Xiangnan Liu, Meiling Liu, Ling Wu

**Affiliations:** School of Information Engineering, China University of Geosciences, Beijing 100083, China; 3004160002@cugb.edu.cn (T.L.); liuml@cugb.edu.cn (M.L.); wuling@cugb.edu.cn (L.W.)

**Keywords:** heavy metal stress, time-series, remote sensing phenology, MODIS and Landsat, ensemble model, feature selection

## Abstract

Heavy metal pollution in crops leads to phenological changes, which can be monitored by remote sensing technology. The present study aims to develop a method for accurately evaluating heavy metal stress in rice based on remote sensing phenology. First, the enhanced spatial and temporal adaptive reflectance fusion model (ESTARFM) was applied to blend Moderate Resolution Imaging Spectroradiometer (MODIS) and Landsat to generate a time series of fusion images at 30 m resolution, and then the vegetation indices (VIs) related to greenness and moisture content of the rice canopy were calculated to create the time-series of VIs. Second, phenological metrics were extracted from the time-series data of VIs, and a feature selection scheme was designed to acquire an optimal phenological metric subset. Finally, an ensemble model with optimal phenological metrics as classification features was built using random forest (RF) and gradient boosting (GB) classifiers, and the classification of stress levels was implemented. The results demonstrated that the overall accuracy of discrimination for different stress levels is greater than 98%. This study suggests that fusion images can be utilized to detect heavy metal stress in rice, and the proposed method may be applicable to classify stress levels.

## 1. Introduction

The present situation of heavy metal pollution in China is severe, which not only poses a serious threat to agricultural product quality, and thus harms human health, but also leads to social instability [1]. The national soil survey jointly released by the Ministry of Land and Resources and the Ministry of Environmental Protection showed that the over-standard rate of heavy metals in cultivated land was 19.4%. According to statistics, more than 10 Mt of grain are lost every year in China due to heavy metal pollution [2,3]. Rice is the crop with the largest area in China and cadmium (Cd, and Cd hyperaccumulation), as one of the most toxic elements to the human body, has become an important limiting factor on rice quality. Cd has been extensively studied and its hazard to human health is regularly reviewed by international organizations such as the World Health Organization (WHO). Cd accumulates primarily in the kidneys, leading to renal tubular dysfunction and the formation of kidney stones. High intake of Cd can lead to disorder in calcium metabolism, which results in osteomalacia, osteoporosis, and painful bone fractures. There is sufficient evidence that long-term exposure to Cd increases the risk of lung cancer, kidney cancer, and prostate cancer [4,5]. In recent years, China has identified the prevention and control of heavy metal pollution in cultivated land as the key task of development planning [6,7]. Therefore, it is of great significance to monitor heavy metal stress accurately and timely.

Traditional methods for monitoring heavy metal contamination, such as sample collection and laboratory analysis, can obtain heavy metal concentrations accurately, and lay a good theoretical foundation for studying the remote sensing mechanism of heavy metal pollution. However, it is difficult to establish a wide range of contamination monitoring in the short term, and meanwhile, long-term field trials add complexity and cost. Remote sensing (RS), with the merits of huge observation scope, cost-effectiveness, real-time effects and non-destruction, has become an efficient method for monitoring heavy metal pollution in crops [8,9,10,11].

As the change in a plant’s physiological elements, such as chlorophyll content, cell structure, and water or nitrogen content, can be monitored by the reflection spectrum, some research on heavy metals in rice have been carried out to identify the relationship between sensitive spectral characteristics and heavy metal concentrations or physiological factors by establishing empirical or semi-empirical models. However, the spectral data only reflected the stress state of one or several growth stages, which may lead to randomness. Still some researchers established the assimilation framework based on RS and the crop growth models to achieve the dynamic monitoring of heavy metal stress from the aspects of physiological functions. In such studies, the dry weight of crop roots (WRT) is generally considered as a good indicator to evaluate stress levels, yet deficiency exists in reduced sensitivity to heavy metal stress as roots age. Many studies have shown that heavy metal cadmium (Cd) poisoning can induce short leaves, decreased chlorophyll content, serious yellowing, and delayed maturity. In addition, Cd stress affects the water content of rice by reducing water absorption [12,13,14,15]. Combining spectral, temporal, and spatial information, the phenological features extracted using remote sensing technology reflect continuous growth and the stress state of rice throughout the entire growth stage. Time-series and phenological characteristics have been proved to be useful to monitor heavy metal stress in rice [10,16,17,18]. Phenological detection is implemented through analysis of time series of remotely-sensed images [19,20,21,22]. One of the most commonly used datasets in phenology studies is collected by the Moderate Resolution Imaging Spectroradiometer (MODIS) sensors on the Terra and Aqua satellites. However, it is challenging to detect changes at small scales or in heterogeneous landscapes using moderate resolution data. Landsat data are insufficient from a temporal perspective due to cloud contamination or revisit cycle limitation. An alternative solution is to develop a high spatial and temporal resolution multi-source remote sensing data fusion method [22,23,24,25]. Zhu et al. proposed the enhanced spatial and temporal adaptive reflectance fusion model (ESTARFM) algorithm to make use of the correlation to blend multi-source data and, meanwhile, to minimize systemic biases and enhance the accuracy of predicting reflectance in changing, heterogeneous landscapes [26].

Since the 1970s, many researchers have recognized the potential of multi-temporal satellite observations to provide information about the phenological development of natural vegetation and crops. VIs are designed to take advantage of spectral reflection/absorption characteristics of plants to enhance the vegetation signal and allow dependable spatial-temporal inter-comparisons in terrestrial photosynthetic activity and canopy structural variations [27,28]. Therefore, the time-series data of vegetation index with distinct seasonal rhythm are suitable for phenological study [29]. In previous phenological-based stress detection studies, indices reflecting greenness information (e.g., Normalized Difference Vegetation Index, NDVI; Enhanced Vegetation Index, EVI) or moisture information (e.g., Normalized Difference Water Index, NDWI) were used independently, which may ignore some critical information. Therefore, it is important to monitor heavy metal stress by combining greenness and moisture information. Because the original temporal vegetation index (VI) profiles include various noise components, such as aerosols and bidirectional reflectance distribution factors, noise reduction or fitting techniques, such as the Savitzky-Golay (S-G) filter, Fourier analysis, and asymmetric Gaussian and double logistic fitting are applied to reconstruct the original VI time series before application [30,31]. Some researchers have compared these smoothing models, and revealed that the Whittaker smoother (WS) had a consistently superior performance in most cases [32,33,34]. From the smoothed VI time profiles, phenological periods can be estimated by the derivation method [35,36], but this method is unable to accurately determine the transplanting period, and the algorithm that combines rice transplanting signals and agronomic rules provides a new method for the extraction of phenological stages [37,38,39]. Previous studies established one or several phenological indicators for heavy metal stress monitoring, making it difficult to effectively exploit phenological information. Moreover, the discriminative features have been constructed manually. Thus, we need to probe for the phenological characteristics and apply some automatic feature extraction methods to stress detection. In most cases, the NDVI or EVI was used empirically for estimation, which rarely determined the optimal VIs for stress estimation. Therefore, the key is to select the optimal discriminative features from this large feature set. Küçük et al. [40] applied support vector machine recursive feature elimination (SVM-RFE) to select the top four features for phenology monitoring. The result showed the feasibility of machine learning (ML) algorithms in feature selection. At present, ML algorithms, such as support vector machines (SVM), decision trees (DT) and random forests (RF) have been utilized in classification [41,42,43,44,45], and the distinction of heavy metal stress can be considered a classification problem. The goal of classification problems is to find a model that best predicts the desired data. The ensemble method involves multiple models and fuses them into a final model, which generally achieves better predictions [44].

Based on the analysis above, a scheme for stress evaluation was explored in this work. In addition to mining the annual temporal change in VIs using time series analysis methods, we took advantage of crop greenness, moisture condition, and corresponding stress characteristics during the whole growth period. In the following sections, we gave a detailed description of our stress level classification method and present its application in Zhuzhou City, Hunan Province, China using fusion images generated by MODIS and Landsat time-series datasets.

## 2. Materials

### 2.1. Study Area

**Figure 1:**
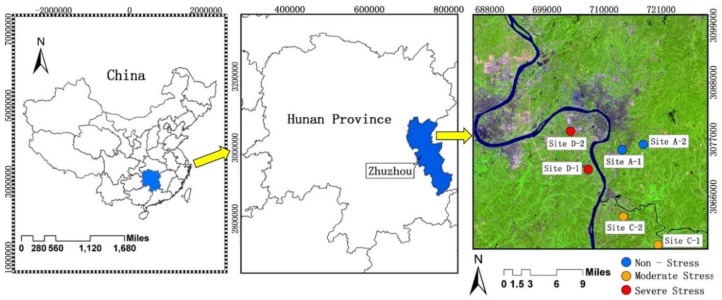
Location map of the six study sites in Zhuzhou, Hunan Province, China.

Our study area, ranging between 26°–28° North and 112.5°–114° East, is located in Zhuzhou City, Hunan Province, China. The region has a subtropical monsoon humid climate with abundant rainfall, and adequate light and heat for rice growth, and the predominant soil type is red soil with sufficient organic matter (2–3%). These climatic and soil conditions promote high yields of rice, making it an important grain commodity base. However, large areas have been contaminated by heavy metals due to sewage irrigation from Xiang Jiang River [46,47]. Previous studies have shown that Cd is the predominant pollutant in paddy soils that are watered from the Xiang Jiang River, which contains industrial wastewater [46,47]. The contaminated paddy fields have resulted in rice growth that is stressed by heavy metals. Six study sites were selected in the research area (Figure 1). According to the content of the heavy metal Cd, the heavy metal stress levels in rice at these six sites were classified as non-stress, moderate stress and severe stress, respectively (Table 1). The same rice type (Boyou 9083) is under intensive cultivation patterns in all sites to ensure adequate irrigation and sufficient fertilizers in paddy fields without pests, weeds, or other environmental issues.

### 2.2. Data Preparation

The experiment was carried out during the entire rice growing stages in 2013. The data included remote sensing images and field measurements. We downloaded Landsat 8 OLI (Operational Land Imager) and Landsat 7 ETM+ (Enhanced Thematic Mapper Plus) Level-2 surface reflectance products in the study area from early May to late October from the United States Geological Survey (USGS) EarthExplorer (http://earthexplorer.usgs.gov/), and then performed layer stacking and regional clipping on Landsat data. In addition, due to the failure of the Landsat-7 airborne scan line corrector (SLC) in May 2003, gapfill processing was required for the ETM+ images. The MOD09A1 product is 8-day composite surface reflectance data with spatial resolution of 500 m. Zhuzhou City is covered by two tiles (h28v06 and h27v06) of MOD09A1 data. We obtained the two tiles from National Aeronautics and Space Administration (NASA) (https://ladsweb.modaps.eosdis.nasa.gov/) and conducted image mosaic and reprojection to MODIS data using Modis Reprojection Tool (MRT) software. Furthermore, in order to implement the ESTARFM algorithm, the order of MOD09A1 data layer stacking was adjusted to match the order of Landsat data. The ESTARFM algorithm is the key to generating synthetic images, which requires at least two pairs of fine- and coarse-resolution images obtained on the same date, as well as a set of coarse resolution images for prediction. The algorithm implementation includes four components: (1) Search similar pixels based on two ETM+ or OLI images at different times; (2) compute the weights (Wi) of all similar pixels; (3) decide the conversion coefficients (Ci) by linear regression; and (4) predict the fine-resolution reflectance from MOD09A1 image on the desired date by Wi and Ci. Moreover, according to the pixel value interpretation of quality assessment (QA) information distributed by USGS, we masked out the pixels that might be affected by instrument artifacts or cloud contamination for subsequent research [48,49].

We conducted a field survey in six study sites of the research area. In each study site, the latitude and longitude coordinates at the center of each sample plot were measured by GPS. In each plot, the rice samples and soil samples were simultaneously collected and preserved in sample bags and soil boxes, and then sent to the laboratory for analysis of the physicochemical characteristics. Each sampled plot contained four subplots, and we sampled 30 g of soil and a whole rice plant in each subplot. The soil of four subplots in each sample plot was mixed for heavy metal measurement; that is, the average heavy metal content of soil samples in each plot was taken as its heavy metal content. The heavy metal content in the soil was analyzed at the Chinese Academy of Agricultural Sciences. 0.5 g of soil samples were put into polytetrafluoroethylene digestion tube with 5 mL of hydrochloric acid and 10 mL of nitric acid (excellent grade), and then placed overnight. On the next day, 2 mL of hydrofluoric acid and 2 mL of perchloric acid were added to digest the samples by heating. Finally, 2–3 mL of liquid was transferred to a 50 mL plastic volumetric flask for cooling and constant volume, and the metal content was determined by inductively coupled plasma mass spectrometer ICP-MS (Model: Agilent 7900, Agilent Technologies, Santa Clara, CA, USA).

## 3. Methods

**Figure 2:**
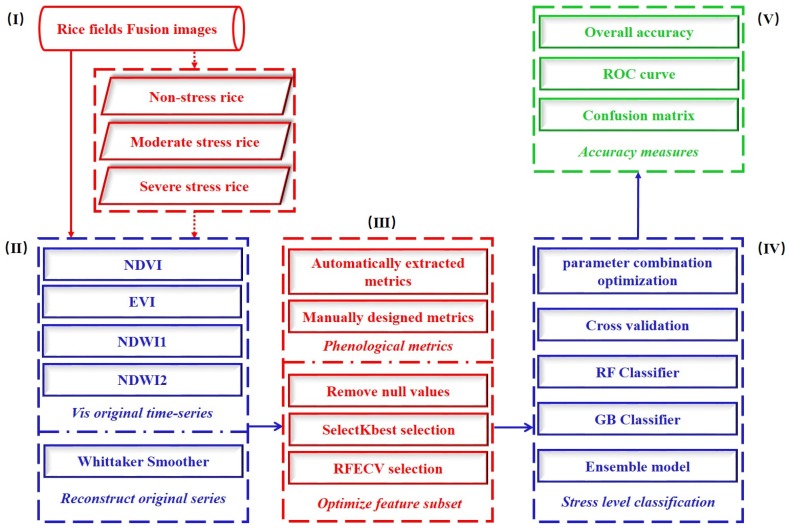
Overview of methodology.

A method for accurately monitoring rice under heavy metal stress at the region scale was proposed (Figure 2). It focuses on the following procedures: (i) calculate vegetation indices and create time series; (ii) design phenological parameters based on changes in rice phenology under heavy metal stress using Matlab and Tsfresh package; (iii) select an optimal phenological feature subset from the original feature set, and establish an ensemble model based on machine learning algorithms to classify heavy metal stress levels in rice; and, (iv) evaluate the accuracy of classification results.

### 3.1. Construction of VI Time Series for Phenological Analysis

Images with cloud cover of less than 30% in the research area were used as inputs of the ESTARFM algorithm, and a series of fusion images was obtained. Depending on the QA bands, we masked out the pixels that exhibited instrument artifacts or cloud contamination, and removed the images with more bad-quality rice pixels. Finally, we obtained 22 images, including original Landsat images and the synthetic images, which were combined to create time series data in chronological order. For each image, we calculated four vegetation indices—(a) NDVI, (b) EVI, (c) NDWI(1) and (d) NDWI(2)—using the land surface reflectance values of the blue ($\rho_{blue}$), green ($\rho_{green}$), red ($\rho_{red}$), NIR ($\rho_{nir}$), SWIR1 ($\rho_{swir1}$), and SWIR2 ($\rho_{swir2}$) bands. NDVI and EVI were selected for their performance in detecting canopy greenness. We also applied NDWI(1) and NDWI(2), which are sensitive to leaf water and soil moisture [50,51,52]. The spectral indices were calculated using the following equations:

(1) $$\mathrm{NDVI}=\left(\rho_{nir}-\rho_{red})/(\rho_{nir}+\rho_{red}\right)$$

(2) $$\mathrm{EVI}=2.5*\left(\rho_{nir}-\rho_{red})/(\rho_{nir}+6*\rho_{red}-7.5*\rho_{blue}+1\right)$$

(3) $$\mathrm{NDWI}\left(1\right)=\left(\rho_{nir}-\rho_{swir1})/(\rho_{nir}+\rho_{swir1}\right)$$

(4) $$\mathrm{NDWI}\left(2\right)=\left(\rho_{nir}-\rho_{swir2})/(\rho_{nir}+\rho_{swir2}\right)$$

The Whittaker smoother is based on penalized least squares [53]. It fits a discrete series to discrete data and puts a penalty on the roughness of the smooth curve [54]. The fitting effect, *Q*, depends on the fidelity to original data, the roughness of smoothed data, *R*, and the smoothing parameter, *k*. The aim of penalized least squares is to find the series, *z*, that minimizes *Q*. Suppose a noisy series y. The smoother *z* is, the more it will deviate from *y*. The larger the parameter *k*, the stronger the influence of *R* on the goal *Q*. Some researchers have found that setting the *k* value to 2 has a higher fitting accuracy [55,56]. Accordingly, we set the *k* value to 2 in this study. The smoothing program was run in Matlab software. The algorithm is extremely fast, provides continuous control over smoothness with only one parameter, and interpolates automatically. The filtering efficiently removed the negatively biased noise present in the original data, while preserving the overall shape of the curves showing vegetation growth and development [56]. It has shown a high potential for remotely sensed time series filter and phenological study [32,56]. Therefore, the WS was chosen in this research.

### 3.2. Designing Phenological Metrics from Seasonal Patterns of VIs

We extracted four key phenological phases, that is, rice transplanting, tillering, heading, and maturity. For each growth cycle, the heading date was first identified based on maximum value. In the transplanting phase, rice paddy fields are a mixture of water and green rice plants, and NDWI values are larger than NDVI or EVI values; specifically, here we recognized the transplanting signals using the criteria NDWI > EVI or NDWI > NDVI [37,38,39]. We selected the date with the maximum value of the first derivative to identify the active tillering phase, and the date that corresponded to zero of the second derivative was considered to be the maturity stage [35,36]. Studies [12,13,14,15,16,17] have shown that: (1) when the rice is exposed to heavy metal stress (e.g., from Cd), enzyme required for chlorophyll formation is inhibited, and chlorophyll content decreased, resulting in chlorosis symptoms in rice, which performed in the NDVI/EVI time-series is the reduction of maximum and minimum NDVI/EVI values; (2) the toxicity of heavy metals can influence the ability of organs to accept and convert photosynthetic products, resulting in reduced growth rate and the length of growth season; (3) the toxicity of heavy metals can also lead to other phenological changes, such as postponed heading date, decreased water content, delayed greening and maturation. Therefore, for unstressed rice, the EVI or NDVI values at heading dates and magnitude of the EVI or NDVI change might be larger than for stressed rice, and the time range from the end of transplanting to the heading date might be shorter compared to stressed rice. Moreover, changes of water content in non-stressed rice during the rice growth period are generally different from those in stressed rice. We built phenological indicators based on the phenological differences under heavy metal stress.

We calculated the annual average, maximum and minimum values of VIs, and extracted some phenological parameters with reference to TIMESAT [57]. Furthermore, two growth stages were determined based on the estimated phenological periods: the early growth stage was defined as the period from estimated transplanting to heading date, and the late growth stage was defined as the period from the estimated heading date to the maturity date. Some phenology-based indicators were designed during the early or the late growth stage. Reed et al. [27] verified that these indicators may not necessarily directly correspond to conventional, ground-based phenological events, but show strong coincidence with expected phenological characteristics. It has been proved that phenology can be a practical indicator for heavy metal stress in rice plants [16,17]; the significance of these indicators lies in the possibility to map out phenological changes in vegetation [58]. Basic details of the phenological signatures are listed in Table 2. To further mine the hidden phenological information in VI time series, we extracted features automatically by time-series feature extraction method. Time-series feature extraction is a time-consuming process because researchers have to consider the multifarious algorithms of signal processing and time-series analysis for identifying and extracting meaningful features from time series. The Python package Tsfresh accelerates this process by combining 63 time-series characterization methods, and automatically calculates a large number of time series characteristics (794 time series features by default) [59,60]. The Tsfresh package has a built-in filtering procedure, which mathematically removes the extracted null-value features [59,60]. In this study, we automatically obtained thousands of features employing the Tsfresh package.

### 3.3. Classification of Heavy Metal Stress Levels in Rice

The hyper-dimensional feature space consisted of thousands of remote sensing features. It was necessary to apply an optimal feature selection strategy to this space to reduce redundancy and computation, and the optimal feature subsets inherited the original physical/mathematical meanings of the features [61]. The normalized feature selection scikit-learn exposes feature selection routines as objects that implement the transform method [62]. SelectKBest, as a class in the scikit-learn library, can be used for feature selection. We applied SelectKBest to obtain a preliminary feature subset. The analysis of variance (ANOVA) F-value is the default function of SelectKbest, which has been used in statistical discriminant analysis. The F-value is an statistic which estimates the significance of variables participating discriminant efficiency [63,64]. We implemented feature ranking based on the F-value, the large F-values were preferred, and then went backwards, until the desired number of features were obtained. Then, we executed feature selection through recursive features elimination with cross-validation (RFECV). RFE involves selecting features by recursively considering smaller and smaller sets of features, that is, acquiring the importance of each feature and pruning the least important features from the current feature set [40]. This procedure is recursively repeated on the pruned set until the desired number of features to select is eventually reached. RFECV performs RFE in a cross-validation loop to find the optimal features. As a result, an optimal feature subset was built for the classification task.

We conducted classification and regression tree (CART) classification on the Landsat 8 OLI image on 17 September 2013, extracted the farmland areas, and then selected rice pixels based on a field survey and Google Earth. Consequently, 1838 rice pixels were obtained for research. Previous work has shown that using boosting and bagging ensemble classifiers achieved greater accuracy than using single classifiers, and was more stable and robust to noise in the training data [44,65]. The random forest (RF) classifier is a good example of the bagging method, and the gradient boosting (GB) classifier is based on the boosting method. We built an ensemble model for the classification of heavy metal stress levels using RF and GB classifiers, that is, firstly, we respectively exploited the two classifiers (RF and GB) to calculate the probabilities that each rice pixel belongs to each stress level, then averaged the probabilities obtained by these two classifiers and took these average values as the final probabilities of the pixel. In addition, in order to set the parameter values with the best classification effect in the classifiers, we exhaustively considered all parameter combinations and systematically traversed a variety of parameter combinations, and finally determined the best performance parameter values through cross-validation.

### 3.4. Accuracy Assessment

We randomly split the original data (1838 rice pixels) into two categories (training set and test set) according to the ratio of 7:3. Training data were used to fit classification models, and testing data were used to evaluate the classification accuracy through the trained model. Cross-validation is a good performance evaluation method in the case of limited data. Here we used 3-fold cross-validation to divide the training set into three equal parts, two of which were used as training sets, the remaining one was used as a verification set. We obtained the prediction results for each validation set, then averaged the correct rates obtained from these three validation sets as the standard to measure the performance of the trained model. In the end, the distinction accuracy for heavy metal stress levels was assessed on the basis of indicators, including the overall accuracy, confusion matrix and the receiver operating characteristic (ROC) curve. Overall accuracy indicates the proportion of all predictions that are correct [66]. A confusion matrix gives a visual representation of the quality of the discrimination results [67]. The ROC is a graphical plot which illustrates the performance of classification model as the discrimination threshold is varied [68].

## 4. Results

### 4.1. Rice Growth Trajectory under Different Heavy Metal Stress

**Figure 3:**
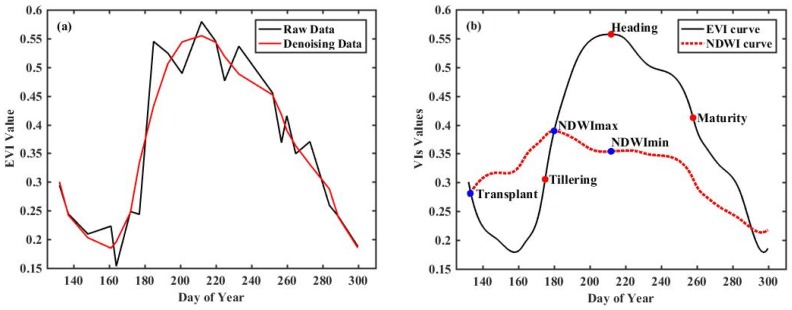
(**a**) The raw EVI data and the reconstructed EVI time-series curve fitted by WS. (**b**) The key phenology-based dates extracted from temporal profiles of EVI and NDWI(1).

Figure 3a presents the fitting results of the WS model with smoothing parameter *k* = 2. It can be seen that the reconstructed curve is smooth and fits the original data well, which conforms to the growth and development of rice. The statistical measures, such as mean absolute deviation (MAE), agreement coefficient (AC), root mean square error (RMSE) and correlation coefficient, were utilized to evaluate the WS model. These four parameters were calculated for each rice pixel between the original data and fitted data, which are 0.0413, 0.8594, 0.0414 and 0.9516, respectively. The smaller the RMSE is, the better the fit would be, and the large correlation coefficient value indicates a close fit. Hence, the WS model performed well in filtering remote sensing time series. As shown in Figure 3b, during a rice growing cycle, the EVI values during the rice transplanting and tillering period could be considerably lower compared to NDWI values due to irrigation, and generally increased with tillering, then reached their peaks in the heading phase. Approximately 50 days after transplanting, most of the rice paddy fields were fully covered by the rice canopy, and NDWI values were lower than those of EVI. At the end of the maturation stage, the rice canopy has lower leaf and stem moisture content and more senescent leaves. Therefore, the EVI values gradually declined as rice was harvested. Similar to the EVI temporal profile, we observed a slight decrease before maturation, and a remarkable decrease after maturation, in the NDWI time series curve. Hence, the parameters based on single or combined variations of EVI/NDVI and NDWI during specific phenological periods could be designed to indicate the growth status of rice.

**Figure 4:**
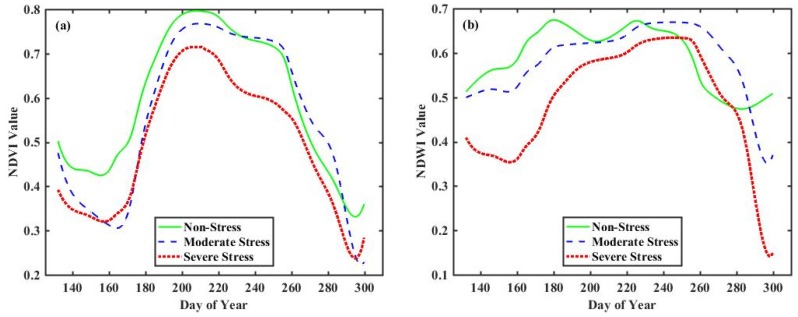
Schematic diagram of changes in (**a**) greenness and (**b**) moisture under different heavy metal stress levels.

The curves in Figure 4a,b were plotted by averaging the VI values of rice pixels at the corresponding study site, which showed growth characteristics of rice, as well as the difference information about rice growth under different heavy metal stress levels. The NDVI and NDWI curves generally follow a rising trend in the early growth stage, and decline in the late growth stage. The distinct shape of the curves under different heavy metal stress are mainly reflected in the following aspects. First, the maximum EVI and NDWI values in severe curves are smaller than those in moderate stress and non-stress curves. Second, for the NDWI curves, the values of non-stress curves are larger compared to those of moderate stress and severe stress in the early growth stage, and the severe stress curve drops the most among the three curves in the late growth stage. Third, for the NDVI curves, the values in severe curves are generally smaller than those in other two curves during the whole rice growth period. In addition, the phenological parameters extracted from the non-stress curve, such as base level and seasonal integral, were greater than those from moderate and severe stress curves. However, it is still impossible to accurately distinguish the three stress levels based on this obvious difference information. Thus, it is necessary to extract more implicit phenological information from time-series data.

### 4.2. Identification of Optimal Number of Feature Subset

**Figure 5:**
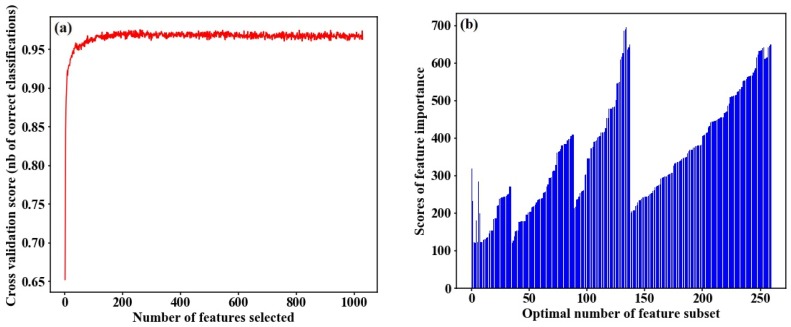
(**a**) Overall classification accuracy using the RF classifier with respect to different feature set obtained by the RFECV algorithm. (**b**) The importance scores of optimal number of features set.

**Figure 6:**
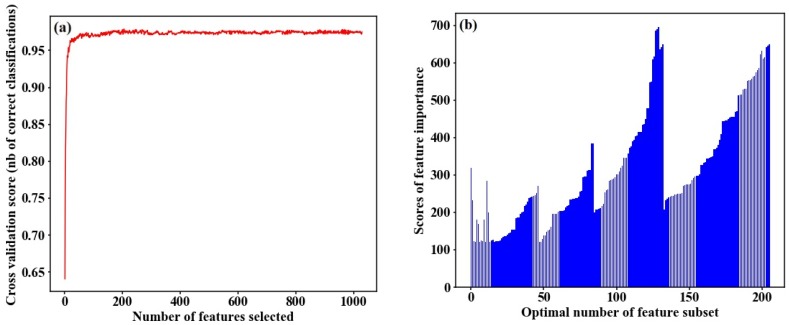
(**a**) Overall classification accuracy using the GB classifier for different feature set obtained by the RFECV algorithm. (**b**) The importance scores of optimal number of features set.

A total of 9528 features were automatically extracted from the VI time-series data, and 3677 features were obtained by removing the null values from the Tsfresh package. After SelectKBest screening, the features with importance scores greater than 120 were chosen to create a preliminary feature subset, including 1029 manually and automatically built signatures. We applied RF-RFECV and GB-RFECV algorithms to respectively analyze the contribution of the features to classification accuracy, as shown in Figure 5 and Figure 6. Figure 5a and Figure 6a show the overall classification accuracies for RF-RFECV and GB-RFECV in which the features are incrementally added to the classification analysis. When the number of features equals 1, accuracy is the lowest. Along with the increase of features, the classification accuracy significantly increased until the characteristic number is greater than 200. We can see from Figure 5a that the accuracy value is around 0.96 as the number of selected features is between 200 and 1029. The maximum correct identification rate reaches 0.97, corresponding to the characteristic number of 260. Nevertheless, the classification accuracy obtained by using all the 1029 features was 0.96. Therefore, instead of using all the features in classification, a higher accuracy can be achieved with using only 260 features. A similar trend is also presented in Figure 6a, and we selected 206 optimal features with the GB-RFECV algorithm. Figure 5b and Figure 6b provide detailed information about the impact of selected features on the classification result. We can see the optimal feature subset selected by RF-RFECV and GB-RFECV; the features with importance scores greater than 150 account for 95% and 87%, respectively. Some features, such as NDVI growth ratio and moisture movement during tillering to heading, scored 139.73 and 145.83, respectively. However, these features were filtered out by the RFECV algorithm in order to reduce the redundancy between relevant features, so will not be discussed further. Additionally, the indicator corresponding to the maximum value, 696.41, which was automatically extracted by Tsfresh, is greater than the contribution of manually established indicators.

### 4.3. Classification Results of Stress Levels

**Figure 7:**
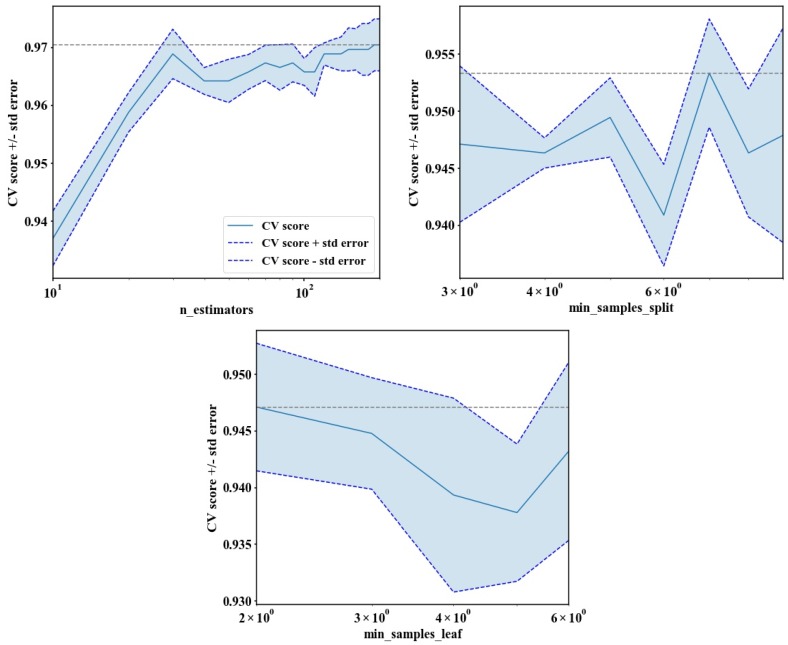
The assessment results under different parameter values.

**Figure 8:**
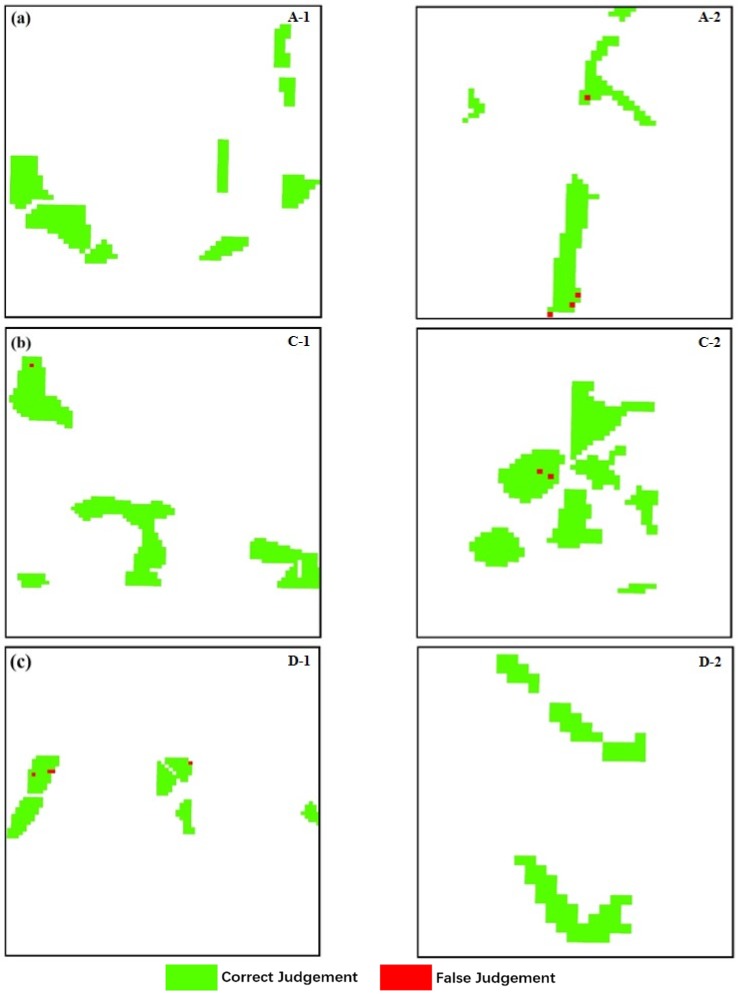
Classification results for (**a**) non-stress, (**b**) moderate stress, and (**c**) severe stress areas.

Because n_estimators, min_samples_split and min_samples_leaf are critical input parameters in the ensemble model, the optimal parameter values must be determined. The ranges of parameters were set as follows: (n_estimators: 10–200), (min_samples_split: 3–9), (min_samples_leaf: 2–6). Figure 7 presents the effect of different parameter values on the accuracy of the model; that is, different parameter values leads to different results, and the model accuracy does not rise as parameter values increases. We traversed all important parameter combinations, and computed the accuracy through cross-validation. Finally, the important parameters in RF were determined as follows: (n_estimators: 120), (min_samples_split: 9), (min_samples_leaf: 2); and the important parameters in GB were determined as follows: (n_estimators: 110), (max_depth: 3). Of the 1286 randomly selected training samples, there are 372 pixels in non-stress rice, 680 pixels in moderate stress rice, and 234 pixels in severe stress rice, which were used for building the ensemble model. The 3-fold cross validation was applied to evaluate the quality of the ensemble model, with an accuracy of 0.988, indicating that we obtained a good-quality ensemble model that can be used for classification of stress levels. At 30-m spatial resolution, maps of the ensemble model with optimum parameter combination applied at the regional scale were generated (Figure 8). Figure 8a–c show the discrimination results for non-stress, moderate stress, and severe stress, respectively. Among them, the green part is the area that belongs to positive judgment; the red part is the area that belongs to misjudgment. Comparing the classification results of different stress levels in Figure 8, it is found that there are different degrees of misclassification for the three stress levels, however, the ensemble model can achieve good results on the whole.

### 4.4. Validation of Discrimination Results

**Figure 9:**
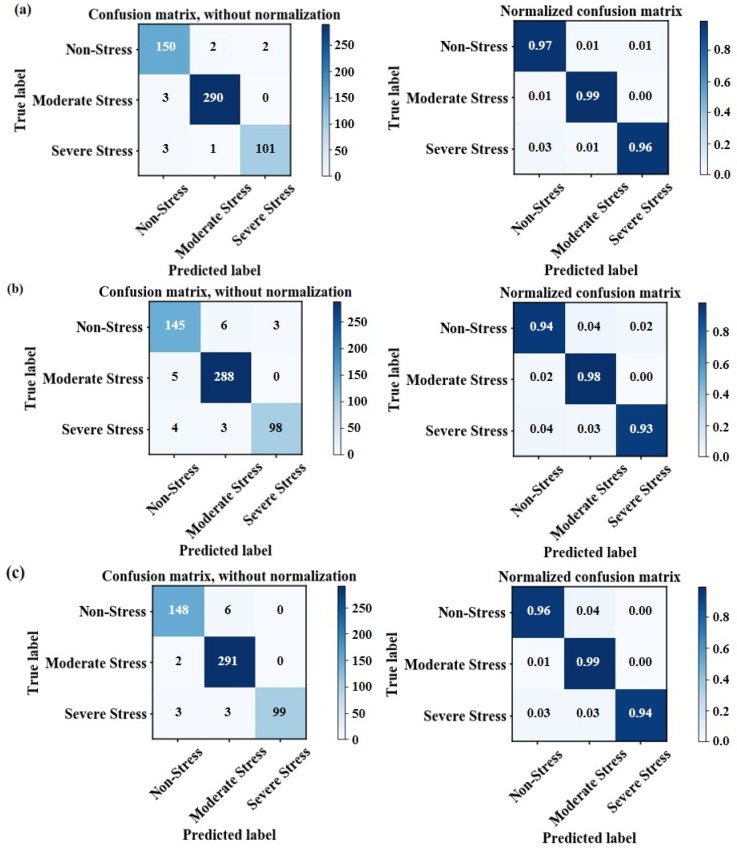
Confusion matrix between estimated and actual stress categories based on (**a**) the ensemble model, (**b**) the RF classifier and (**c**) the GB classifier.

**Figure 10:**
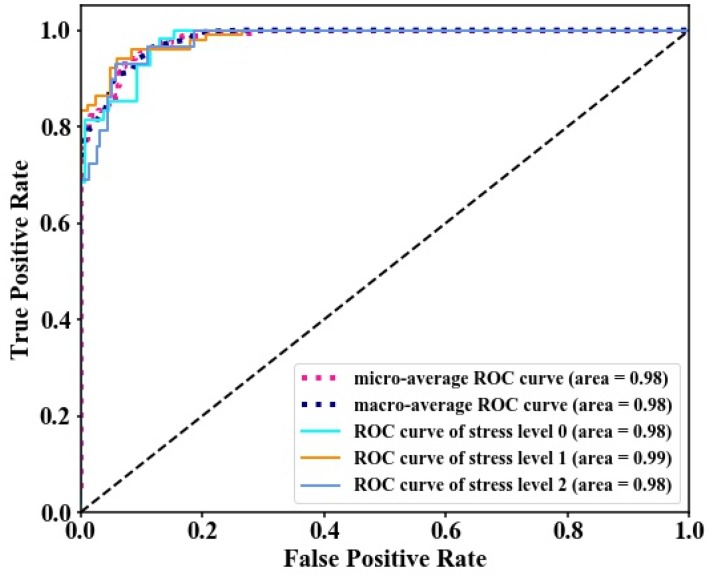
The evaluation results based on ROC curves and AUC values.

According to the discrimination model established above, an accuracy assessment was conducted base on test datasets, which included a total of 552 randomly selected rice pixels. The confusion matrix that compared the predicted results and actual results was generated to evaluate classification accuracy. Among the 154 non-stress rice pixels, 150 were identified as non-stress rice (97% agreement). For 293 moderate-stress rice pixels, 290 were identified as moderate-stress rice (99% agreement). From Figure 9a, the severe stress region had 101 pixels for positive judgment and 4 pixels misjudged, with a positive rate of 96%. An overall accuracy of 98.01% was obtained when compared with the actual dataset. Furthermore, we can see from Figure 9 that the discrimination effect of the ensemble model is better than that of using RF and GB separately. The discrimination accuracy for non-stress and severe-stress level rice increased by 1–3% and 2–3% respectively, and the overall accuracy for different stress categories showed an improvement of approximately 1–2%. Figure 10 presents the ROC and area under curve (AUC) values to evaluate the classification results. The y-axis represents the rate at which a pixel is a positive class and is predicted to be a positive class. The *x*-axis represents the rate at which a pixel is a negative class and is predicted to be a positive class. The closer the AUC value is to 1, the better the differentiation effect. The AUC value here reached 0.98 and 0.99, which means we obtained a high accuracy for classifying different heavy metal stress categories.

## 5. Discussion

**Figure 11:**
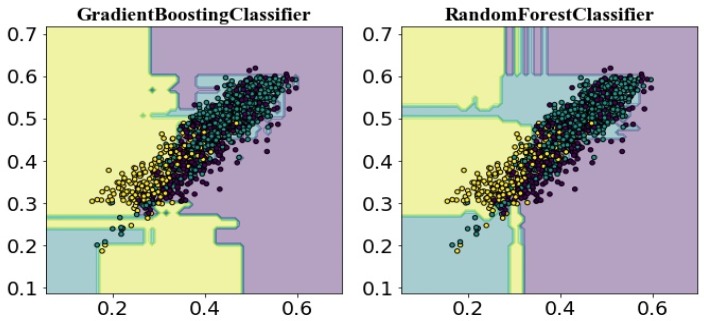
The decision boundaries of GB and RF classifiers.

We obtained a satisfactory performance in classifying heavy metal stress levels in rice, which can be partly attributed to the effectiveness of spatio-temporal fusion images for detecting rice stress and the selection of high-quality rice pixels. In addition to high-quality rice pixels, the accuracy in reconstructing VI time series is dependent on the de-noising effect. The WS model performed well in de-noising, as well as being in accordance with the growth characteristics of rice, with RMSE and correlation coefficient of 0.0414 and 0.9516, respectively. The extracted four key phenological periods, namely transplanting, tillering, heading and maturation, which conformed to rice phenology and transplanting characteristics, can be used to establish phenological indicators to detect heavy meal stress in rice. Other features extracted by the Tsfresh package were also applied to this research. Therefore, the capability and necessity of extracting multi-dimensional features were discussed. We plotted the decision boundaries of three stress levels based on only two features (NDVI and EVI amplitude); these two features with high importance scores were used here. From Figure 11, we can see that the two features were able to discriminate different heavy metal stress to a certain extent. However, many moderate stress pixels are mixed with unstressed pixels. Figure 5 showed that classification accuracy was around 0.96 based on all 1029 features for RF or GB classifiers. Compared with using all features in the classification, high accuracy cannot be maintained using only two features (Table 3). Hence, we acquired manually and automatically designed features to fully reflect phenological information in VI time-series, and achieved accurate discrimination for heavy metal stress levels using high-dimensional phenological features.

Manually designed indicators were based on the critical phenological periods, which would leave out important phenological information at other stages. The time-series curves of different stress levels contain visible and invisible difference information; without Tsfresh, all complex and implicit characteristics would have to be calculated by hand. The extracted features based on Tsfresh can be used to build models that perform classification tasks on the time series [59]. Therefore, the automatically extracted characteristics were able to compensate for the limitations of the manually designed indicators to some extent. Further quantitative analysis was conducted by comparing the classification results obtained using both the manually and automatically designed features with those obtained using either the manually or automatically designed features only, and the classification accuracy reached 0.972, which was greater than the individual accuracies, which were 0.918 and 0.963, respectively. In summary, the best stress level estimation results were obtained using the manually and automatically designed features together. Due to the negative impact of some non-essential features on classification results, we carried out primary selection by the SelectKBest program and further screening by the REFCV algorithm. As seen in Section 4.2, the optimal feature subset produced the highest classification accuracy. The feature selection method employed in this study not only developed faster machine learning models, but also achieved the best discrimination results.

**Figure 12:**
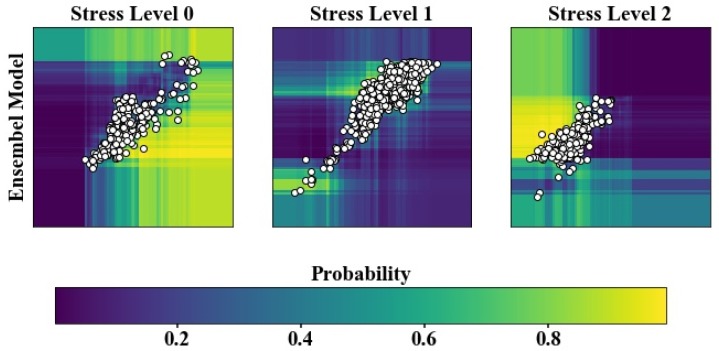
The probabilities that rice pixels belong to different stress classes for the ensemble model.

In order to allow the classification model to have stronger generalization and achieve better results, we combined GB and RF classifiers and performed the classification process by voting. That is, the probabilities of each rice pixel belonging to different stress categories were predicted by GB and RF, and then the predicted results of the two classifiers were averaged. We took the above two features (the amplitudes of NDVI and EVI) as examples to plot the classification probability for the ensemble model. Figure 12 presents the averaged probabilities that rice pixels belong to different stress categories. Yellow cells represent the area with the highest probability. All points falling in the yellow area are considered to have the largest probabilities, with the stress category corresponding to the maximum probability taken as the final category. Section 4.4 described the classification capacity of these three classifiers in detail. Furthermore, Figure 9 demonstrated in an intuitive way that the ensemble model had the best performance in determining boundaries of the stress classes. It has been verified that the classification ability of ensemble model was greater than that of the RF and GB classifiers.

## 6. Conclusions

This study applied the ESTARFM to fuse Landsat data with MODIS data, and then generated multi-temporal synthetic images to detect Cd stress on rice growing at regional scale. A scheme was built by considering the estimation of heavy metal stress levels as a classification problem. Firstly, 3677 features were obtained based on manual establishment and automatic extraction. Secondly, optimal feature selection was employed, in which 260 signatures of the RF classifier and 206 signatures of the GB classifier were selected using the proposed feature selection method. Finally, an ensemble model using RF and GB classifiers was utilized due to its capability for improving accuracy. The result of the scheme described above showed that: (1) The synthetic images offer promise for the accurate detection of rice under Cd stress; (2) the feature selection model is effective, with the stress level estimation accuracy achieved using the optimal features shown to be higher than that using all features; (3) the distinction accuracy based on a combination of the features extracted manually and automatically was higher than that based on either manual establishment or automatic extraction; and, (4) the discrimination accuracy obtained by the ensemble model was higher than that considering the RF and GB classifiers separately.

In future studies, the response mechanism of rice phenology to the concentration of heavy metals will be further explored. Additionally, it is necessary to consider the crop varieties, various cultivation practices, climatic conditions, and growth environments in different areas, and take these characteristics as important input parameters of the model. The classification model of heavy metal stress should be optimized by conducting validation at more sites.

## Figures and Tables

**Table 1:** Heavy metal Cd concentration of the six study sites.

Study Sites	Geographic Location	Cd Concentration in Soil	Background Value	National Quality Standard	Pollution Level
A-1	113°12′ E 27°47′ N	0.84	1.43	0.3–1.0	None
A-2	113°10′ E 27°47′ N	0.84			None
C-1	113°14′ E 27°37′ N	2.25			Moderate
C-2	113°10′ E 27°40′ N	2.31			Moderate
D-1	113°06′ E 27°45′ N	3.27			Severe
D-2	113°04′ E 27°49′ N	3.54			Severe

Note: The unit of Cd concentration is mg/kg. The quality standard of soil environment is used to evaluate pollution levels. Cd background value was obtained from the Hunan Institute of Geophysical and Geochemical Exploration, China.

**Table 2:** List of phenological signatures manually established in this study.

Feature Category	Parameters	Number of Parameters
Phenological signatures	Annual average, maximum and minimum values of VIs	18
	Base level, seasonal amplitude, seasonal integral, seasonal length and growth ratio	10
	VIs(heading)-VIs(maturity)	6
	VIs(heading)-VIs(tillering)	6
	T(heading)-T(transplantend);	2
	(NDWImax-NDWImin)/(VI(heading)-VI(maturity))	2
	(NDWImax-NDWImin)/(VI(heading)-VI(tillering))	2
Total		46

Note: VIs refers to NDVI, EVI, NDWI(1), NDWI(2), NDVI^2^+NDWI(2)^2^ and EVI^2^+NDWI(1)^2^. VIs(heading), VIs(maturity), VIs(tillering) refers to the VIs values in heading, maturity and tillering date respectively. VI(heading), VI(maturity) and VI(tillering) refers to the EVI or NDVI values in heading, maturity and tillering date respectively. T(heading) and T(transplantend) refers to the dates in heading and the end of transplant respectively.

**Table 3:** Classification accuracy of RF and GB classifiers based on two phenological features.

Assessment Measures	Random Forest	Gradient Boosting
Overall Accuracy	67.57%	66.12%
Correct-Judgement Rate for Non-Stress Rice	51.94%	48.70%
Correct-Judgement Rate for Moderate-Stress Rice	79.52%	78.16%
Correct-Judgement Rate for Severe-Stress Rice	57.14%	58.10%
